# Safety and Efficacy of Direct Oral Anticoagulants Versus Vitamin K Antagonists for Cerebrovascular Ischemic Outcomes in Non‐Valvular Atrial Fibrillation: A Systematic Review and Meta‐Analysis

**DOI:** 10.1002/clc.70308

**Published:** 2026-04-17

**Authors:** Anam Nasir, Naqash Anwar, Abdullah Bin Kamran, Ayesha Muhammad, Ali Haider, Muhammad Mubashar, Fatima Tariq, Mostafa Helou, Besher Shami

**Affiliations:** ^1^ University of South China Hengyang Hunan China; ^2^ Department of Medicine Rawalpindi Medical University Rawalpindi Pakistan; ^3^ Nanchang University Nanchang China; ^4^ Gannan Medical University Ganzhou China; ^5^ University of Aleppo, Faculty of Medicine Aleppo Syria; ^6^ Berkshire Medical Center Pittsfield Massachusetts USA

## Abstract

**Background:**

Atrial fibrillation (AF) is a major cause of thromboembolic events, including ischemic stroke and transient ischemic attack (TIA). While Vitamin K antagonists (VKAs) have long been used for stroke prevention, Direct Oral Anticoagulants (DOACs) have emerged as potential alternatives due to improved pharmacologic profiles and safety. This systematic review and meta‐analysis aimed to compare the efficacy and safety of DOACs versus VKAs in patients with non‐valvular AF, with a focus on cerebrovascular ischemic outcomes.

**Methods:**

A comprehensive search of PubMed, ClinicalTrials.gov, and Cochrane Library was performed in accordance with PRISMA 2020 guidelines. Randomized controlled trials and comparative observational studies reporting cerebrovascular events, major bleeding, and all‐cause mortality were included. Pooled risk ratios (RR) with 95% confidence intervals (CI) were calculated using a random‐effects model.

**Results:**

Eleven studies encompassing 814 716 patients were included. DOAC use was associated with a significantly lower risk of recurrent cerebrovascular ischemic events compared with VKAs (RR 0.83, 95% CI 0.78–0.88, *p* < 0.0001). The risk of major bleeding was also reduced with DOACs (RR 0.77, 95% CI 0.71–0.82, *p* < 0.0001). All‐cause mortality was similar between groups (RR 1.02, 95% CI 0.34–3.13), though sensitivity analysis excluding one heterogeneous study favored DOACs (RR 0.79, 95% CI 0.63–0.98).

**Conclusion:**

In patients with non‐valvular AF, DOACs demonstrate superior efficacy in reducing cerebrovascular ischemic outcomes and lower bleeding risk compared to VKAs, with comparable mortality outcomes. These findings support current guidelines recommending DOACs as first‐line anticoagulation for stroke prevention in AF.

## Graphical abstract

Direct Oral Anticoagulants (DOACs) were associated With lower rates of cerebrovascular ischemic events and major bleeding compared With Vitamin K antagonists in non‐valvular atrial fibrillation, With similar mortality. These findings support DOACs as the preferred anticoagulant strategy for stroke prevention in contemporary clinical practice.

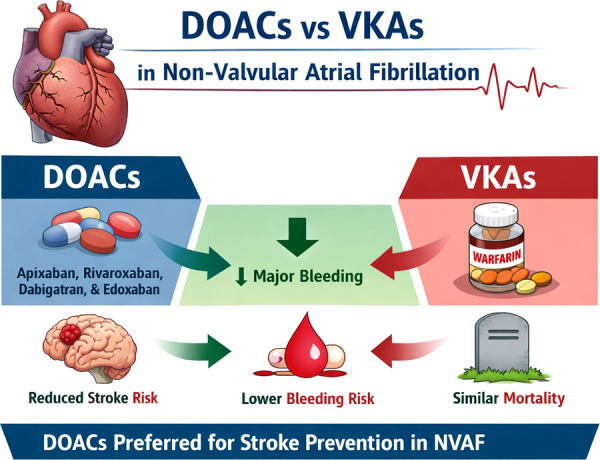

## 1 Introduction

Atrial fibrillation (Afib) is the leading cause of sustained cardiac arrhythmias in clinical practice, affecting 37.6 million people annually. Prevalence further increases as the population gets older due to the rise in cardiovascular diseases [1]. The presence of AF raises the probability of thromboembolic events, with ischemic stroke accounting for up to 20% of all ischemic events, and is significantly correlated with high morbidity and mortality [2]. Thrombus formation in Afib occurs due to a mix of blood stasis, endothelial damage, and hypercoagulability; thrombi usually arise in the left atrium within the left atrial appendage [3].

Vitamin K antagonists (VKAs) like warfarin and acenocoumarol were the mainstay of anticoagulation in Afib for decades [4]. The effectiveness of warfarin in decreasing the risk of ischemic stroke and systemic embolism to about one‐third that of placebo has been recognized successfully by several clinical trials held in the late 20th century [5]. However, VKAs have several disadvantages that stem from the nature of their mechanism of action: a very narrow therapeutic index requiring close monitoring of INR, vast variability between patients due to diet and genetic factors, and a great frequency of drug‐drug interactions [6]. The slow effect, along with regular dose changes, leads to low patient compliance. All these issues have made the search for more effective, safe, and convenient alternatives even more urgent.

Direct Oral Anticoagulants (DOACs) such as dabigatran, rivaroxaban, apixaban, and edoxaban are one such alternative [7]. In the case of dabigatran, the direct action is against thrombin (Factor IIa) [8]; on the other hand, rivaroxaban, apixaban, and edoxaban are selective inhibitors of Factor Xa [9]. By avoiding the Vitamin K‐dependent pathway, DOACs achieve rapid onset of action, fixed dosing without routine monitoring, and fewer pharmacologic interactions. These pharmacokinetic advantages have led to the widespread adoption of DOACs for stroke prevention in Non‐valvular AF (NVAF) [10].

The efficacy and safety of DOACs have been established by several landmark randomized controlled trials (RCTs). The RE‐LY trial [11] was a double‐blind study involving more than 18 000 patients with NVAF, where dabigatran was compared to warfarin. The study found that dabigatran at a dose of 150 mg twice daily was able to decrease the risk of stroke and systemic embolism significantly without the complication of major bleeding. The ROCKET‐AF trial [12] showed that rivaroxaban was the same as warfarin in terms of stroke and systemic embolism prevention, but there was a much lower incidence of intracranial bleeding. The ARISTOTLE trial [13] added to the evidence by demonstrating that not only did apixaban reduce stroke and systemic embolism, but also there was a considerable drop in major bleeding and all‐cause mortality in comparison to warfarin. These landmark studies collectively steered in a new era of anticoagulation practice, resulting in the global acceptance of DOACs as the primary treatment choice in guidelines issued by major organizations like the European Society of Cardiology (ESC, 2020) and the American Heart Association (AHA, 2019).

Despite these advances, the comparative safety and efficacy of DOACs versus VKAs, particularly regarding cerebrovascular ischemic outcomes such as stroke and transient ischemic attack (TIA), remain a subject of ongoing investigation. RCTs with a high number of participants give sound evidence for wide NVAF populations, but their specific inclusion criteria often cut off old people, those with many diseases, and others who have had strokes or TIAs. Analysts are looking back in time and using real‐world data to suggest that there may be differences in outcomes based on patient demographics, renal function, and medicine use that are taken at the same time. For example, old or renal‐impaired patients may undergo drug clearance alterations and have different bleeding risks. Besides that, TTR, which indicates the quality of VKA management, has been used to explain why some studies report different results in different regions and through different organizations.

Cerebrovascular ischemic events, encompassing both stroke and TIA, represent one of the most devastating complications of AF. Effective prevention depends on balancing thromboembolic risk, assessed by the CHA₂DS₂‐VASc score, against the risk of bleeding [14]. While DOACs have demonstrated lower rates of intracranial hemorrhage, data regarding cerebrovascular ischemic outcomes in patients with AF remain inconsistent [15].

Given the variability among published studies, an updated analysis of existing data is required to guide clinical practice and future recommendations. Accordingly, the present systematic review and meta‐analysis were conducted to evaluate the comparative safety and efficacy of DOACs and VKAs in patients with NVAF, with emphasis on cerebrovascular ischemic outcomes. Data from RCTs and retrospective cohort studies published within the past decade were analyzed to determine whether DOAC therapy provides improved protection against recurrent ischemic outcomes with an acceptable safety profile. The objective of this analysis is to produce a consolidated assessment of anticoagulant performance, thereby contributing to evidence‐based management of stroke prevention in AF.

## 2 Methods

### 2.1 Search Strategy

An extensive search was carried out by the authors in PubMed, Clinicaltrials.gov, and Cochrane Library, for studies published in English, starting from inception to September 2025, to identify all existing literature, without any restrictions of time. PRISMA guidelines [16] were followed for the systematic review. Medical subject heading (MeSH) terms as well as free‐text terms were utilized in each database. The keywords used included “Acenocoumarol”, “Sinthrome,” “Rivaroxaban,” “5‐chloro‐N‐(((5S)−2‐oxo‐3‐(4‐(3‐oxomorpholin‐4‐yl)phenyl)−1,3‐oxazolidin‐5‐yl)methyl)thiophene‐2‐carboxamide,” “Warfarin,” “4‐Hydroxy‐3‐(3‐oxo‐1‐phenylbutyl)−2H‐1‐benzopyran‐2‐one,” “N‐((2‐(((4‐(aminoiminomethyl)phenyl)amino)methyl)−1‐methyl‐1H‐benzimidazol‐5‐yl)carbonyl)‐N‐2‐pyridinyl‐beta‐alanine,” “Dabigatran,” and “Atrial Fibrillation” among many others, with no restrictions on study design. This was followed by a manual search for relevant literature from selected studies and published reviews.

### 2.2 Study Selection and Inclusion Criteria

Reviewers ABK and AN reviewed the titles, abstracts, and full texts of all the articles after the search independently. The inclusion criteria were set to [1]: Randomized, controlled, parallel trials, or comparative observational studies [2]; Intervention group receiving Direct‐acting Oral Anticoagulants, including Rivaroxaban, Dabigatran, Apixaban, and Edoxaban, and the control group receiving VKAs: Warfarin, Acenocoumarol; and [3] available outcomes on Cerebrovascular events, Bleeding incidence, and mortality. Studies were included based on reporting cerebrovascular ischemic outcomes, regardless of whether prior stroke or TIA was a baseline inclusion criterion. Case reports, editorials, review articles, and studies not published in English were excluded.

Any disagreements between the two authors were resolved through a group consensus and a third adjudicator (N.A.).

### 2.3 Data Extraction and Quality Assessment

Data pertaining to all the study outcomes, along with relevant study characteristics, were extracted and entered into Microsoft Excel. The recorded data included study authors, design, sample size, population, population age, gender, treatment and control interventions, Baseline CHA₂DS₂‐VASc score, Baseline incidence of Hypertension, Diabetes Mellitus, Coronary artery disease, as well as total follow‐up period for each study.

The analysis was carried out in Review Manager v5.4.1. Cochrane's Risk of Bias (ROB) Assessment tool in Review Manager v5.4.1 was used for randomized, controlled trials, whereas the Newcastle‐Ottawa scale was used for the observational retrospective cohorts [17].

### 2.4 Outcomes and Analysis

The primary safety and efficacy outcome was Cerebrovascular events defined as incidents of TIAs and Ischemic Stroke incidents labelled in various studies, and Major Bleeding events, as well as All‐cause mortality, respectively.

Risk ratios (RRs) were calculated and pooled, via the Mantel‐Haenszel method, for the overall estimate of DOACs' efficacy through the number of patients suffering from a TIA or ischemic stroke. Random effects models were prepared for all outcomes. Heterogeneity across the studies was assessed using the Higgins *I* ^2^ statistic along with Cochran's *Q* test. Significance testing was two‐sided, and *p* < 0.05 was considered statistically significant.

## 3 Results

### 3.1 Search Results

**Figure 1:**
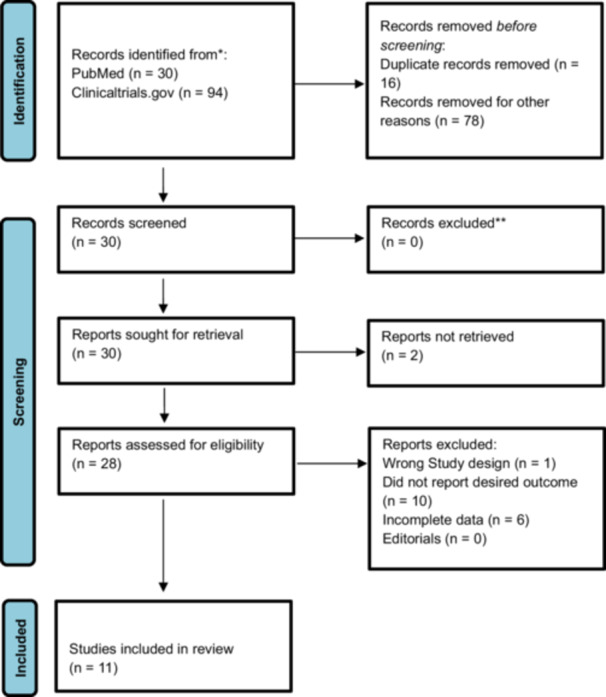
PRISMA flowchart for screening.

A total of 124 records were initially identified across the databases. After removal of 16 duplicates, 108 records remained for title and abstract screening. Of these, 30 articles were selected for full‐text review. Following full‐text assessment, 11 studies met the inclusion criteria and were included in the final analysis. The PRISMA flowchart is shown in Figure 1.

### 3.2 Study Characteristics

11 studies were included in the analysis. The patients were divided into two groups. The control group in all studies received either Warfarin or Acenocoumarol, with target INR mostly specified as 2.0−3.0. The treatment group received one of the 4 Direct‐acting Oral Anticoagulants: Apixaban, Rivaroxaban, Dabigatran, or Edoxaban. The study characteristics are shown in Table 1.

**Table 1:** Study characteristics.

Study author	Study year	Study design	Region	Study duration	Patient population	Mean age		DOACs used (Dosage)	VKAs (Target INR)
						DOACs	VKAs		
Vlachos [18]	2017	RCT	Greece	June 2013 to December 2016	Patients who underwent catheter ablation for symptomatic paroxysmal or persistent AF	57.69 ± 11.7	58.91 ± 10	Dabigatran, Apixaban, or Rivaroxaban (Adjusted)	Warfarin, Acenocoumarol (2.0−3.0)
Russo [19]	2019	Retrospective Cohort	Italy	July 2013 to January 2018	Patients with nonvalvular AF and a history of bioprosthetic heart valve replacement	64.1 ± 9.2	73.3 ± 6.4	Apixaban 2.5/5 mg, Rivaroxaban 15/20 mg, Dabigatran 110/150 mg, or Edoxaban 30/60 mg	Warfarin, Acenocoumarol
Clara [20]	2021	Retrospective Cohort	Spain	November 2011 to December 2015	Patients of atrial fibrillation	72.30 ± 11.30	74.82 ± 9.58	Apixaban, Dabigatran, or Rivaroxaban	Acenocoumarol
Mugnai [21]	2017	Retrospective Cohort	Belgium and Italy	June 2013 to September 2015	Patients with drug‐resistant paroxysmal, persistent and long‐standing persistent AF who underwent Pulmonary Vein isolation by Cryo‐balloon ablation	62.8 ± 9.7	58.6 ± 11.3	Dabigatran 150 mg Twice daily, Apixaban 5 mg twice daily, Edoxaban 60 mg once daily, or Rivaroxaban 20 mg once daily	Warfarin, Acenocoumarol (2.0–3.0)
Montero‐Balosa [22]	2025	Retrospective Cohort	Spain	January 2012 to December 2020	Patiients over 40 years of age, with ICD‐9 or ICD‐10 diagnosis of AF given initial treatment with oral anticoagulants	73.2 ± 10.6	74.5 ± 9.9	Apixaban, Dabigatran, Rivaroxaban, or Edoxaban	Acenocoumarol
Hori [23]	2014	RCT	Japan	June 2007 to January 2010	Patients aged 20 years or older with non‐valvular AF	72.5	72.5	Rivaroxaban	Warfarin
Costa [24]	2021	Retrospective Cohort	USA	November 2010 to December 2019	Patients who had non‐valvular AF and Diabetes type 2 newly started on Rovaroxaban or Warfarin on or after November 1st 2011	70 ± 11	73 ± 10	Rivaroxaban	Warfarin
Guimarães [25]	2020	RCT	Brazil	April 2016 to July 2019	Adults (≥ 18 years of age) who had permanent, paroxysmal, or persistent atrial fibrillation or flutter and a bioprosthetic mitral valve and who were receiving (or planning to receive) oral anticoagulation for thromboembolism prophylaxis	59.4 ± 2.4	59.2 ± 11.8	Rivaroxaban 20 mg 0nce daily	Warfarin (2.0–3.0)
Cappato [26]	2014	RCT	Italy	October 2012 to September 2013	Patients aged 18 years or older scheduled for elective electrical or pharmacological cardioversion	64.9 ± 10.6	64.7 ± 10.5	Rivaroxaban 20 mg 0nce daily	Warfarin (2.0–3.0)
NCT02061748 [12]	2017	Retrospective Cohort	USA	October 2010 to April 2013	Patients diagnosed with non‐valvular atrial fibrillation (NVAF) in the real‐world setting using the Humana population	73.2 ± 8.6	74.8 ± 8.2	Dabigatran 7 mg or 150 mg	Warfarin 1–10 mg
Ako [27]	2025	Retrospective Cohort	Japan	March 2013 to December 2021	Patients diagnosed with AF	85.6 ± 4.1	85.5 ± 4.3	Apixaban 2.5 mg or 5 mg orally	Warfarin

The studies were published between the year 2014 and 2025. There were 4 randomized controlled trials and 11 cohorts. The general data extracted from the studies is shown in Table 2.

**Table 2:** General study data. Data are either Mean ± SD or Median (Range).

Study	Sample size	DOACs					VKAs					Follow‐up
		Baseline CHA2DS2‐VASc score	Baseline HAS‐BLED score	Hypertension	DM	CAD	Baseline CHA2DS2‐VASc score	Baseline HAS‐BLED score	Hypertension	DM	CAD	
Vlachos	474	1.26 ± 1.2	0.75 ± 0.8	41.10	8	3.80	1.40 ± 1.1	0.79 ± 0.8	49.30%	6.60%	10.30%	90 days
Russo	464	2.5 ± 2.2	2.4 ± 1.1	35.50	18.5	8.5	3.4 ± 2.5	3.5 ± 1.5	45.4	28.2	15.3	26.8 ± 2.3 months
Clara	41,560	3.64 ± 1.82	2.87 ± 1.24	77.18	31.57	16.97	3.83 ± 1.65	2.93 ± 1.17	80.51%	36.03	18.29	4 years
Mugnai	454	1.78 ± 1.29	1.21 ± 0.52	64	13	8	1.53 ± 1.35	0.87 ± 0.56	50	11	11	Not mentioned
Montero‐Balosa	150,949	3.4 ± 1.71	2.3 ± 1.0	76.7	24.9	Not mentioned	3.60 ± 1.61	2.5 ± 1.0	78.3	37.01	Not mentioned	Not mentioned
Hori	1278	3	Not mentioned	77.5	42.1	37.7	3	Not mentioned	77.9	43.4	34.9	30 days
Costa	83182	3.1 ± 1.2	1.4 ± 0.8	91.6	Not mentioned	10.4	3.5 ± 1.2	1.6 ± 0.8	90.2	Not mentioned	12.2	Till occurrence of any outcome of interest, end of EHR activity or 31 December 2019
Guimarães	1005	2.7 ± 1.5	1.6 ± 0.6	61.6	14.8	1.6	2.5 ± 1.3	1.6 ± 0.9	59.8	12.7	1.4	12 months
Cappato	1504	*N/A*	Not mentioned	65	20.3	9	*N/A*	Not mentioned	68.7	20.5	6.6	Not mentioned
Ako	495347	3.1 ± 1.1	Not mentioned	39.2	17	14.5	3.1 ± 1.1	Not mentioned	38.1	16.5	13.9	*N/A*
NCT02061748	38499	Not mentioned	Not mentioned	Not mentioned	Not mentioned	Not mentioned	Not mentioned	Not mentioned	Not mentioned	Not mentioned	Not mentioned	*N/A*

**Figure 2:**
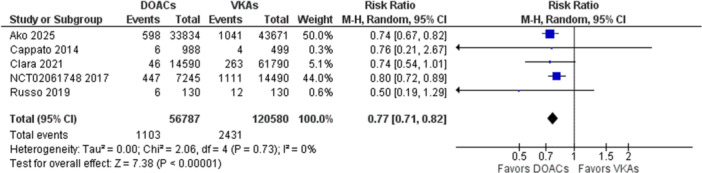
Forest plot for cerebrovascular ischemic events in DOACs versus VKAs.

The total patient population added up to 814,716 patients. The follow‐up time varied from 30 days to 4 years. The DOAC and VKA groups were mostly equally matched in baseline characteristics in all studies. The Newcastle‐Ottawa scale was used to estimate ROB in Retrospective Cohorts, while Cochrane's ROB Tool (ROB2) was used for RCTs, as shown in Figure 2.

### 3.3 Cerebrovascular Ischemic Events (TIA/Stroke)

**Figure 3:**
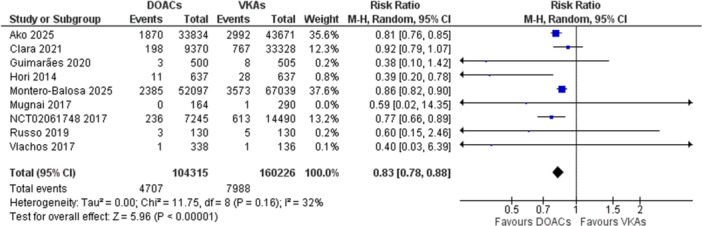
Forest plot for major bleeding risk in DOACs versus VKAs.

The primary outcome for efficacy was the risk of cerebrovascular ischemic events, including TIA and Stroke. Nine studies included in the analysis mentioned this outcome. Seven thousand nine hundred eighty‐eight patients (4.98%) in the VKA group experienced a TIA or an ischemic stroke, while only 4707 patients (4.51%) in the DOACs group had an ischemic event. The pooled effect showed DOACs to be significantly more efficacious in reducing risk of a TIA and Ischemic stroke (RR 0.83, 95% CI [0.78, 0.88]). There was no significant heterogeneity noted in the overall effect (Z = 5.9, *p* < 0.0001). Figure 3 shows the forest plot for the outcome.

### 3.4 Major Bleeding Risk

Risk of any major bleeding—defined as fatal bleeding or symptomatic bleeding in a critical area, or organ, or bleeding causing a decrease in hemoglobin level of 2 g/dL or leading to transfusion of two or more units of whole blood or red cells, was taken as an outcome to evaluate safety between the two groups. Five studies reported the outcome. A random‐effects model was prepared to pool the outcome. The overall effect showed a significantly reduced risk of a major bleed in the DOAC group as compared to patients using Warfarin or Acenocoumarol (RR 0.77, 95% CI [0.71, 0.82]). There was no heterogeneity among the studies. The overall effect was significant (*Z* = 7.38, *p* < 0.0001).

### 3.5 All‐Cause Mortality

**Figure 4:**
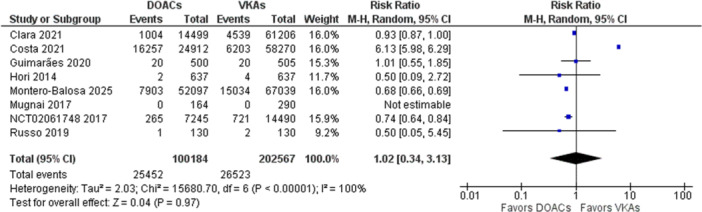
Forest plot for all‐cause mortality in DOACs versus VKAs.

Another outcome of safety was all‐cause mortality. This outcome was reported by 8 out of the 11 studies included in the analysis. 25.40% of the patients in the DOAC group died during the study period, while in the VKA group, only 13.09% of the patients suffered from mortality. This is reflected in the overall effect as well, which shows a slight increase in risk of all‐cause mortality in the DOAC group (RR 1.02, 95% CI [0.34, 3.13]). Although the overall effect was insignificant (*Z* = 0.04, *p* = 0.97). The forest plot for all‐cause mortality is shown in Figure 4.

**Figure 5:**
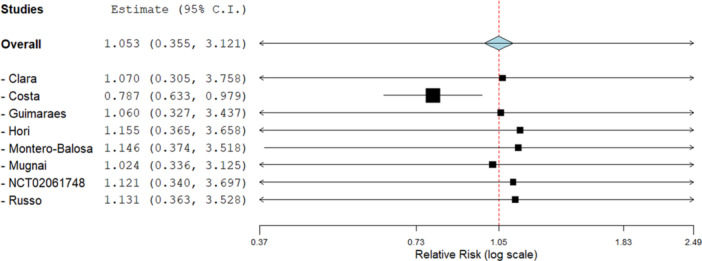
A leave‐one‐out analysis for all‐cause mortality.

There was significant heterogeneity noted in the outcome (*I* ^2^ = 100%). To rectify this heterogeneity, a leave‐one‐out analysis was performed. This showed the study by Costa et al. [24] to be the main cause of heterogeneity. Leaving this study out of the analysis resulted in a significant reduction in the heterogeneity as well as a drastic reduction in the risk of all‐cause mortality in the DOAC group, making it the favorable group (RR 0.79, 95% CI [0.63, 0.98]) compared to the VKA group. Removing the study also resulted in a significant overall effect (*Z* = 2.16, *p* = 0.03). The forest plot for the leave‐one‐out analysis is shown in Figure 5.

## 4 Discussion

This systematic review and meta‐analysis examined the comparative safety and efficacy of DOACs and VKAs in patients with NVAF, with emphasis on cerebrovascular ischemic outcomes. Data extracted from eleven studies, including RCTs and observational cohorts, were used. Three primary outcomes were assessed: the incidence of cerebrovascular ischemic events, the risk of major bleeding, and all‐cause mortality.

Across the included studies, the use of DOACs was associated with a lower risk of recurrent ischemic stroke or TIA when compared with VKAs (RR 0.83, 95% CI 0.78–0.88). This result shows a modest but consistent reduction in cerebrovascular events among patients receiving DOAC therapy. The consistency of this finding aligns with earlier clinical evidence. Ruff et al. [28] demonstrated a similar benefit in their meta‐analysis, reporting that DOACs reduced the risk of stroke or systemic embolism by approximately one‐fifth compared with warfarin. Likewise, a recent network meta‐analysis by Mai et al. [10] showed that DOACs maintained a favorable net clinical outcome across different AF populations, including those with prior stroke.

Although major randomized trials of DOACs have reported subgroup analyses in patients with prior stroke or TIA, these analyses were not uniformly included in the present meta‐analysis due to variability in reporting and study design. Nevertheless, the findings of the current study are consistent with these subgroup analyses and further support the overall benefit of DOACs in AF.

The observed difference between DOACs and VKAs in this outcome likely reflects differences in the mechanism of action and pharmacology. DOACs achieve stable anticoagulation through predictable pharmacokinetics and fixed dosing, minimizing the variability seen with VKAs, which depend on dietary vitamin K intake and frequent INR monitoring. The therapeutic range for VKAs can be narrow, and subtherapeutic anticoagulation increases thromboembolic risk. The absence of significant heterogeneity for this outcome indicates that the reduction in ischemic events with DOACs is reproducible across different populations and study designs.

Major bleeding was consistently lower among DOAC users compared with those on VKAs (RR 0.77, 95% CI 0.71–0.82). This finding supports the growing body of evidence that DOACs are associated with fewer severe bleeding complications, particularly intracranial hemorrhage. Fong et al. [29] noted a comparable reduction in their systematic review, emphasizing that while gastrointestinal bleeding remains a concern, the overall safety profile of DOACs remains superior.

By directly inhibiting a single coagulation factor, DOACs provide a focused anticoagulant effect with fewer variations. In contrast, VKAs interfere with several vitamin K–dependent factors, producing a less predictable response and increasing susceptibility to both over‐ and under‐anticoagulation [30]. Furthermore, DOACs' shorter half‐life and the availability of reversal agents, such as idarucizumab and andexanet alfa, provide additional safety advantages in acute care settings [31]. Evidence from real‐world studies supports these findings. The FRAIL‐AF trial [6] demonstrated that switching older, frail patients from VKAs to DOACs reduced major bleeding without compromising stroke prevention. The low heterogeneity across the studies included in this meta‐analysis further reinforces the reliability of these results.

For overall mortality, no significant difference was detected between the two groups in the primary analysis (RR 1.02, 95% CI 0.34–3.13), and substantial heterogeneity was present (*I*² = 100%). However, sensitivity testing revealed that a single large cohort, Costa et al. [24], accounted for much of this variation. The Costa et al. study likely contributed disproportionately to the observed heterogeneity due to its large sample size and inclusion of a higher‐risk population with type 2 diabetes mellitus. This subgroup is known to have increased cardiovascular risk and mortality, which may have influenced the pooled estimates. Additionally, differences in study design and real‐world data characteristics may have further contributed to variability in outcomes. When this study was excluded, the pooled analysis showed a significant reduction in mortality among DOAC users (RR 0.79, 95% CI 0.63–0.98).

Differences in follow‐up periods, comorbidities, and baseline characteristics likely contributed to the heterogeneity observed in each outcome. Mortality is influenced by multiple factors, including age, cardiovascular status, renal function, and concurrent drug use, which varied considerably among studies. Despite these differences, the adjusted results suggest that DOACs may give a modest survival benefit over VKAs. The findings of the present analysis align with the predominant finding in the literature supporting the clinical advantages of DOACs over VKAs. Several large‐scale trials and meta‐analyses have demonstrated either non‐inferiority or superiority of DOACs in preventing thromboembolic events while reducing bleeding risk. The RE‐LY, ROCKET‐AF, and ARISTOTLE trials collectively established the foundation for DOAC use in AF.

This analysis has several limitations to keep in mind when interpreting the findings. The inclusion of both randomized and retrospective studies introduces methodological variability. While random‐effects modeling was applied, residual confounding cannot be completely excluded. Some observational cohorts may have lacked adjustment for relevant variables such as renal function, body weight, and concurrent antiplatelet therapy, all of which influence anticoagulation outcomes. Outcome definitions were also not uniform across studies. Criteria for “major bleeding” and “ischemic stroke” varied slightly, and this lack of standardization could have influenced pooled estimates. Additionally, individual patient‐level data were unavailable, which prevented stratified analyses by age, sex, or specific DOAC type. Finally, publication bias cannot be ruled out, as studies with neutral or negative results may be underrepresented in the literature.

The inclusion of both RCTs and observational studies introduces methodological heterogeneity. Observational cohorts contributed a substantial proportion of the total patient population, which may introduce residual confounding despite the use of random‐effects modeling.

Overall, the results of this review indicate that DOACs should be considered the preferred anticoagulant class for patients with NVAF, including those at risk of cerebrovascular ischemic events. They offer a lower risk of recurrent stroke and major bleeding compared with VKAs, with at least equivalent survival outcomes. The data also highlight the importance of individualized treatment selection, taking into account renal function, drug interactions, patient adherence, and cost considerations. Ongoing surveillance through national registries and long‐term cohort studies will continue to refine the understanding of DOAC use in diverse patient groups.

## 5 Conclusion

This meta‐analysis demonstrates that DOACs have greater efficacy in reducing cerebrovascular ischemic events and improved safety outcomes, such as major bleeding, when compared with VKAs in patients with NVAF. Mortality outcomes were comparable, though sensitivity analysis suggests a possible advantage favoring DOACs. The results support current guideline recommendations prioritizing DOACs over VKAs for stroke prevention in AF.

## Author Contributions

**Anam Nasir:** writing–original draft, literature review. **Naqash Anwar:** conceptualization, writing–original draft. **Abdullah Bin Kamran:** conceptualization, writing–original draft, formal analysis. **Ayesha Muhammad:** methodology, writing–original draft, writing–reviewing and editing. **Ali Haider:** data collection, writing. **Muhammad Mubashir:** writing–review and editing**. Mostafa Helou:** reviewing and editing, corresponding author. **Besher Shami:** writing–reviewing and editing. **Fatima Tariq:** data collection, methodology, formal analysis.

## Funding

The authors have nothing to report.

## Ethics Statement

The authors have nothing to report.

## Consent

Participants were informed about the use of their data in the analysis for research purposes. No personal data or images were disclosed.

## Conflicts of Interest

The authors declare no conflicts of interest.

## Data Availability

The study data can be requested via email to the first author and shall be made available upon request.
